# Supplementary material for: Neuroanatomical Quantitative Proteomics Reveals Common Pathogenic Biological Routes between Amyotrophic Lateral Sclerosis (ALS) and Frontotemporal Dementia (FTD)
Source: Int J Mol Sci. 2018 Dec 20;20(1):4. doi: 10.3390/ijms20010004 (PMC6337647; doi:10.3390/ijms20010004)
Supplement: Supplementary file 1 [file ijms-20-00004-s001.pdf]

Supplementary Table S1: In this table protein name, uniprot code, gene name p-value and Fold change (FC) for each comparison are shown, for 299 of the 301 significantly regulated proteins found in both comparisons (p-value<0.01, fold change (FC) >+/-0.37) ALS versus control and FTLD-U versus control. Two uncharacterized proteins have been excluded from this list

Protein name	Uniprot	Gene name	p value FTLD-U	FC FTLD-U	p value ALS	FC ALS
**Cytochrome b-c1 complex subunit 7**	P14927	UQCRB	1.534E-03	-1.591E+00	6.005E-04	-1.639E+00
**NADH dehydrogenase [ubiquinone] 1 alpha subcomplex subunit 7**	O95182	NDUFA7	4.127E-04	-9.471E-01	3.467E-05	-1.643E+00
**NADH dehydrogenase [ubiquinone] 1 alpha subcomplex subunit 2**	O43678	NDUFA2	3.230E-04	-9.145E-01	2.113E-04	-1.450E+00
**NADH dehydrogenase [ubiquinone] iron-sulfur protein 5**	O43920	NDUFS5	1.769E-04	-8.829E-01	3.235E-05	-1.007E+00
**ARF GTPase-activating protein GIT1**	A0A0C4DGN6	GIT1	1.306E-03	-8.810E-01	1.115E-03	-7.228E-01
**Methylglutaconyl-CoA hydratase, mitochondrial**	Q13825	AUH	6.097E-04	-7.666E-01	5.619E-06	-1.178E+00
**ADP/ATP translocase 1**	P12235	SLC25A4	6.068E-03	-6.095E-01	3.595E-04	-1.011E+00
**MIC**	J3QTA6	CHCHD6	1.090E-04	-5.913E-01	2.124E-03	-5.948E-01
**MIC**	J3QTA6	CHCHD6	1.090E-04	-5.913E-01	2.124E-03	-5.948E-01
**Protein kinase C and casein kinase substrate in neurons protein 1**	Q9BY11	PACSIN1	3.837E-03	-5.863E-01	3.680E-06	-1.824E+00
**Tubulin polymerization-promoting protein**	O94811	TPPP	6.466E-03	-5.755E-01	6.943E-06	-1.169E+00
**MIC**	C9JRZ6	CHCHD3	2.912E-02	-6.187E-01	2.195E-03	-9.781E-01
**Mitochondrial 2-oxoglutarate/malate carrier protein**	Q02978	SLC25A11	5.385E-03	-5.642E-01	2.067E-04	-6.783E-01
**NADH dehydrogenase [ubiquinone] iron-sulfur protein 6, mitochondrial**	O75380	NDUFS6	6.385E-04	-5.636E-01	2.521E-04	-1.344E+00
**ATP-dependent RNA helicase A**	Q08211	DHX9	6.206E-03	-5.489E-01	5.978E-04	-7.282E-01
**Cytochrome b-c1 complex subunit 6, mitochondrial**	P07919	UQCRH	2.555E-03	-5.462E-01	3.774E-05	-1.112E+00
**ATP synthase subunit e, mitochondrial**	P56385	ATP5I	3.812E-03	-4.960E-01	5.590E-03	-7.127E-01
**D-tyrosyl-tRNA(Tyr) deacylase 1**	Q8TEA8	DTD1	4.453E-03	-4.480E-01	5.201E-04	-6.216E-01

**Isoform 2 of NADH dehydrogenase [ubiquinone] 1 alpha subcomplex subunit 5**	Q16718-2	NDUFA5	8.172E-04	-4.311E-01	3.914E-04	-7.745E-01
**Isoform 2 of Fructose-bisphosphate aldolase A**	P04075-2	ALDOA	2.564E-03	-4.256E-01	7.392E-03	-6.171E-01
**Peptidyl-prolyl cis-trans isomerase NIMA-interacting 1**	Q13526	PIN1	1.130E-03	-3.917E-01	9.565E-06	-9.878E-01
**Mitochondrial import inner membrane translocase subunit Tim13**	Q9Y5L4	TIMM13	1.759E-04	-3.875E-01	2.658E-03	-4.346E-01
**14-3-3 protein eta**	Q04917	YWHAH	4.999E-01	-2.994E-01	5.756E-03	-1.635E+00
**26S protease regulatory subunit 10B**	A0A087X2I1	PSMC6	5.644E-01	1.464E-01	2.276E-03	-8.141E-01
**3-hydroxyacyl-CoA dehydrogenase type-2**	Q99714	HSD17B10	5.735E-01	-7.288E-02	1.485E-04	-6.069E-01
**3-ketoacyl-CoA thiolase, mitochondrial**	A0A0B4J2A4	ACAA2	1.972E-01	-1.698E-01	4.707E-04	-5.742E-01
**A-kinase anchor protein 12**	Q02952	AKAP12	3.603E-01	-9.134E-02	1.614E-03	-4.658E-01
**ATP synthase F(0) complex subunit B1, mitochondrial**	P24539	ATP5F1	2.646E-02	-5.400E-01	2.014E-04	-1.195E+00
**ATP synthase subunit O, mitochondrial**	P48047	ATP5O	2.770E-02	-3.439E-01	1.684E-05	-9.332E-01
**ATP synthase subunit alpha, mitochondrial**	P25705	ATP5A1	3.394E-01	-1.653E-01	3.065E-03	-6.564E-01
**ATP synthase subunit epsilon, mitochondrial**	P56381	ATP5E	2.914E-02	-3.728E-01	1.281E-03	-8.244E-01
**Alpha-internexin**	Q16352	INA	5.864E-01	-1.547E-01	8.300E-03	-9.529E-01
**Ankyrin-2**	I6L894	ANK2	4.074E-01	-2.006E-01	1.965E-01	3.564E-01
**Ankyrin-2**	Q01484	ANK2	1.885E-01	-3.857E-01	7.451E-03	-7.517E-01
**Aspartate aminotransferase, cytoplasmic**	P17174	GOT1	1.663E-01	-2.206E-01	3.226E-04	-8.068E-01
**Aspartate aminotransferase, mitochondrial**	P00505	GOT2	9.215E-02	-4.124E-01	3.229E-03	-8.777E-01
**Beta-adducin**	P35612	ADD2	9.875E-01	-2.137E-03	3.014E-03	-4.535E-01
**Beta-synuclein**	Q16143	SNCB	7.097E-01	-7.728E-02	8.855E-04	-9.587E-01
**Brain acid soluble protein 1**	P80723	BASP1	5.027E-01	-3.308E-01	2.776E-05	-2.879E+00
**CAP-Gly domain-containing linker protein 2**	Q9UDT6	CLIP2	2.721E-01	1.044E-01	3.807E-03	-3.988E-01
**CLIP-associating protein 2**	E7EW49	CLASP2	8.832E-02	2.676E-01	1.164E-04	-6.969E-01
**Calcium/calmodulin-dependent protein kinase type II subunit beta**	Q13554	CAMK2B	6.042E-01	-1.136E-01	2.690E-04	-1.095E+00
**Calcyclin-binding protein**	Q9HB71	CACYBP	9.103E-02	-1.277E-01	6.298E-05	-4.090E-01
**Clathrin light chain B**	P09497	CLTB	1.425E-02	-3.531E-01	9.393E-05	-7.432E-01
**Collagen alpha-2(IV) chain**	P08572	COL4A2	3.343E-02	-6.581E-01	5.603E-03	-9.356E-01

**Creatine kinase U-type, mitochondrial**	P12532	CKMT1A	5.027E-01	-1.007E-01	1.093E-06	-9.984E-01
**Crk-like protein**	P46109	CRKL	1.811E-01	-1.899E-01	1.996E-04	-6.290E-01
**Cyclin-dependent-like kinase 5**	Q00535	CDK5	4.768E-01	-9.335E-02	7.319E-03	-5.475E-01
**Cystatin-C**	P01034	CST3	1.337E-01	-3.599E-01	6.755E-03	-6.734E-01
**Cysteine and histidine-rich domain-containing protein 1**	Q9UHD1	CHORDC1	2.767E-01	-1.879E-01	3.906E-03	-6.403E-01
**Cytochrome b-c1 complex subunit 1, mitochondrial**	P31930	UQCRC1	2.439E-02	-2.676E-01	1.033E-03	-5.904E-01
**Cytochrome b-c1 complex subunit 2, mitochondrial**	P22695	UQCRC2	8.842E-01	3.131E-02	2.903E-04	-1.011E+00
**Cytochrome c oxidase subunit 5A, mitochondrial**	P20674	COX5A	1.070E-01	-2.671E-01	4.390E-05	-1.232E+00
**Cytochrome c oxidase subunit 5B, mitochondrial**	P10606	COX5B	3.017E-02	-5.270E-01	2.304E-04	-1.864E+00
**Cytochrome c oxidase subunit 6A1, mitochondrial**	P12074	COX6A1	6.820E-01	-1.015E-01	1.479E-04	-1.462E+00
**Cytochrome c oxidase subunit 6B1**	P14854	COX6B1	4.828E-02	-3.545E-01	2.623E-05	-9.959E-01
**Dihydrolipoyl dehydrogenase, mitochondrial**	P09622	DLD	2.040E-01	-1.761E-01	1.535E-03	-5.779E-01
**Dihydrolipoyllysine-residue acetyltransferase component of pyruvate dehydrogenase complex, mitochondrial**	P10515	DLAT	9.583E-01	1.003E-02	2.903E-03	-5.745E-01
**Drebrin**	Q16643	DBN1	1.708E-01	-3.295E-01	1.635E-03	-8.918E-01
**Elongation factor 1-alpha 2**	Q05639	EEF1A2	5.839E-02	-7.670E-01	1.248E-03	-1.765E+00
**Endophilin-A3**	Q99963	SH3GL3	7.411E-01	5.451E-02	5.477E-04	-8.744E-01
**Eukaryotic translation initiation factor 5A-1**	I3L504	EIF5A	7.102E-01	-5.812E-02	1.125E-04	-1.150E+00
**Fatty acid-binding protein, heart (Fragment)**	S4R371	FABP3	1.889E-02	-3.356E-01	1.435E-03	-7.746E-01
**Fructose-bisphosphate aldolase C**	P09972	ALDOC	3.917E-01	-1.088E-01	5.564E-05	-7.127E-01
**Fumarate hydratase, mitochondrial**	P07954	FH	3.974E-01	-1.306E-01	8.325E-04	-5.567E-01
**Gamma-enolase**	P09104	ENO2	7.987E-02	-1.478E-01	5.903E-04	-5.851E-01
**Gamma-soluble NSF attachment protein**	Q99747	NAPG	7.597E-02	-1.588E-01	2.599E-04	-5.203E-01
**Gamma-synuclein**	O76070	SNCG	2.869E-01	-1.793E-01	8.657E-05	-7.801E-01
**Glucose-6-phosphate isomerase (Fragment)**	A0A0A0MTS2	GPI	3.983E-01	-1.014E-01	2.509E-03	-4.165E-01
**Glutathione S-transferase kappa 1**	Q9Y2Q3	GSTK1	9.588E-01	1.509E-02	5.204E-03	-1.161E+00

**HIG1 domain family member 1A, mitochondrial**	C9JNU6	HIGD1A	3.272E-01	-2.261E-01	4.101E-04	-1.001E+00
**Heterogeneous nuclear ribonucleoprotein M**	P52272	HNRNPM	7.168E-01	-1.616E-02	1.937E-04	-4.287E-01
**Hyaluronan and proteoglycan link protein 2**	Q9GZV7	HAPLN2	9.009E-02	-5.195E-01	4.286E-05	-1.562E+00
**Hypoxanthine-guanine phosphoribosyltransferase**	P00492	HPRT1	6.308E-01	-7.132E-02	3.900E-03	-5.122E-01
**Immunoglobulin superfamily member 8**	Q969P0	IGSF8	6.157E-02	-2.599E-01	3.015E-04	-7.467E-01
**Interferon-inducible double-stranded RNA-dependent protein kinase activator A**	O75569	PRKRA	4.241E-02	-4.771E-01	4.848E-04	-1.147E+00
**Isoaspartyl peptidase/L-asparaginase**	Q7L266	ASRGL1	8.671E-01	-2.464E-02	7.620E-03	-5.145E-01
**Isoform 2 of Cytoplasmic dynein 1 intermediate chain 1**	O14576-2	DYNC1I1	2.327E-02	-8.297E-01	5.765E-04	-1.651E+00
**Isoform 2 of MIC**	Q16891-2	IMMT	2.545E-01	-1.080E-01	3.325E-05	-4.886E-01
**Isoform 2 of NADH dehydrogenase [ubiquinone] 1 beta subcomplex subunit 4**	O95168-2	NDUFB4	1.728E-02	-4.495E-01	1.082E-06	-2.206E+00
**Isoform 2 of Serine/arginine-rich splicing factor 2**	Q01130-2	SRSF2	4.255E-01	-1.399E-01	2.264E-03	-6.406E-01
**Isoform 3 of Neurochondrin**	Q9UBB6-3	NCDN	8.974E-01	-3.598E-02	1.296E-03	-1.165E+00
**Isoform 4 of Clathrin coat assembly protein AP180**	O60641-4	SNAP91	5.556E-02	-3.402E-01	8.500E-03	-5.470E-01
**Isoform 5 of Actin-binding LIM protein 2**	Q6H8Q1-5	ABLIM2	2.073E-01	-3.341E-01	4.047E-03	-1.110E+00
**Isoform 7 of Cytosolic acyl coenzyme A thioester hydrolase**	O00154-7	ACOT7	8.427E-01	-3.136E-02	1.739E-06	-1.556E+00
**Isoform 9 of Calpastatin**	P20810-9	CAST	4.090E-01	-1.721E-01	3.625E-03	-1.020E+00
**Leucine-rich repeat-containing protein 47**	Q8N1G4	LRRC47	3.200E-02	-3.239E-01	1.376E-04	-7.923E-01
**MAP6 domain-containing protein 1**	Q9H9H5	MAP6D1	3.703E-01	-2.553E-01	1.834E-04	-1.477E+00
**ATP synthase subunit d, mitochondrial**	O75947	ATP5H	3.103E-03	-3.821E-01	4.434E-04	-5.497E-01
**MIC**	C9JRZ6	CHCHD3	2.912E-02	-6.187E-01	2.195E-03	-9.781E-01
**Macrophage migration inhibitory factor**	P14174	MIF	6.514E-02	-5.958E-01	7.627E-03	-8.703E-01
**Malate dehydrogenase, cytoplasmic**	P40925	MDH1	7.469E-01	-3.783E-02	6.571E-05	-6.417E-01
**Malate dehydrogenase, mitochondrial**	P40926	MDH2	2.991E-02	-3.994E-01	1.317E-04	-7.848E-01

**Microtubule-associated protein**	F8W9U4	MAP4	2.742E-01	-2.519E-01	4.201E-03	-9.554E-01
**Microtubule-associated protein**	E7EVA0	MAP4	3.660E-01	2.141E-01	9.383E-01	-1.732E-02
**Microtubule-associated protein 2**	P11137	MAP2	1.979E-01	-4.528E-01	3.386E-03	-1.073E+00
**Microtubule-associated protein 6**	Q96JE9	MAP6	4.823E-02	-3.578E-01	2.345E-05	-1.030E+00
**Microtubule-associated protein RP/EB family member 3**	Q9UPY8	MAPRE3	3.929E-01	3.012E-01	6.312E-03	-9.822E-01
**NADH dehydrogenase [ubiquinone] 1 alpha subcomplex subunit 12**	Q9UI09	NDUFA12	2.913E-02	-5.674E-01	9.288E-05	-1.234E+00
**NADH dehydrogenase [ubiquinone] 1 alpha subcomplex subunit 8**	P51970	NDUFA8	1.421E-02	-3.793E-01	6.289E-06	-1.326E+00
**NADH dehydrogenase [ubiquinone] 1 beta subcomplex subunit 10**	O96000	NDUFB10	2.358E-01	-3.283E-01	1.565E-05	-1.509E+00
**NADH dehydrogenase [ubiquinone] flavoprotein 1, mitochondrial**	P49821	NDUFV1	5.881E-01	-8.649E-02	1.324E-04	-7.454E-01
**NADH dehydrogenase [ubiquinone] flavoprotein 2, mitochondrial**	E7EPT4	NDUFV2	5.104E-01	-1.519E-01	1.537E-03	-8.857E-01
**NADH dehydrogenase [ubiquinone] iron-sulfur protein 4, mitochondrial**	O43181	NDUFS4	1.802E-02	-3.694E-01	8.070E-05	-8.718E-01
**NADH dehydrogenase [ubiquinone] iron-sulfur protein 8, mitochondrial (Fragment)**	E9PPW7	NDUFS8	3.770E-01	-8.931E-02	5.099E-04	-5.568E-01
**Neurocan core protein**	O14594	NCAN	1.309E-02	-8.803E-01	1.447E-04	-1.486E+00
**Neurofilament heavy polypeptide**	P12036	NEFH	1.196E-01	-3.025E-01	1.775E-05	-9.741E-01
**Neurofilament medium polypeptide**	P07197	NEFM	6.672E-01	-6.913E-02	2.338E-04	-7.075E-01
**Neuromodulin**	P17677	GAP43	3.075E-01	-2.426E-01	1.025E-04	-9.534E-01
**Neuronal-specific septin-3**	Q9UH03	SEPT3	4.716E-01	-1.387E-01	3.748E-03	-7.117E-01
**Neuroplastin (Fragment)**	Q9UFM8	DKFZp566H1924	2.161E-02	-3.327E-01	1.408E-05	-9.384E-01
**Nuclear ubiquitous casein and cyclin-dependent kinase substrate 1**	Q9H1E3	NUCKS1	7.015E-01	-6.005E-02	6.802E-03	-4.669E-01
**Oligodendrocyte-myelin glycoprotein**	P23515	OMG	3.570E-01	-1.732E-01	3.424E-04	-8.362E-01
**Optineurin**	Q96CV9	OPTN	1.092E-01	-3.232E-01	1.109E-03	-1.029E+00

**Phosphoglycolate phosphatase**	A6NDG6	PGP	4.097E-01	-2.554E-01	5.051E-03	-1.002E+00
**Prefoldin subunit 6**	O15212	PFDN6	7.431E-01	-4.929E-02	1.418E-03	-6.448E-01
**ProSAAS**	Q9UHG2	PCSK1N	6.737E-02	-6.322E-01	1.901E-03	-1.064E+00
**Programmed cell death protein 5**	O14737	PDCD5	3.410E-01	-1.761E-01	6.664E-03	-6.407E-01
**Protein LOC102724023**	A0A0B4J2D5	LOC102724023	3.695E-01	-6.843E-02	3.063E-05	-5.687E-01
**Pyruvate dehydrogenase E1 component subunit beta, mitochondrial**	P11177	PDHB	6.757E-02	-1.706E-01	7.389E-03	-4.145E-01
**RNA 3'-terminal phosphate cyclase**	O00442	RTCA	3.665E-02	-5.012E-01	2.122E-04	-1.169E+00
**RNA-binding motif protein, X chromosome**	P38159	RBMX	1.566E-01	-2.390E-01	1.607E-03	-9.004E-01
**Rho-related GTP-binding protein RhoB**	P62745	RHOB	4.483E-01	-1.326E-01	5.848E-03	-4.404E-01
**Secretogranin-2**	P13521	SCG2	2.260E-01	-5.114E-01	1.679E-03	-1.429E+00
**Serine/arginine-rich-splicing factor 1**	J3KTL2	SRSF1	4.779E-01	-1.139E-01	6.035E-03	-7.222E-01
**Sideroflexin-1**	Q9H9B4	SFXN1	5.254E-01	-1.230E-01	7.542E-03	-6.787E-01
**Signal recognition particle 14 kDa protein**	P37108	SRP14	9.445E-01	2.428E-02	3.302E-03	-1.369E+00
**Signal transducing adapter molecule 1**	Q92783	STAM	5.392E-02	-2.783E-01	1.914E-04	-6.827E-01
**Single-stranded DNA-binding protein, mitochondrial**	Q04837	SSBP1	1.199E-01	-2.661E-01	5.965E-04	-6.677E-01
**Sodium/potassium-transporting ATPase subunit alpha-1**	P05023	ATP1A1	9.025E-01	-2.696E-02	3.986E-03	-8.406E-01
**Stress-induced-phosphoprotein 1**	P31948	STIP1	5.129E-01	-1.074E-01	1.136E-03	-9.343E-01
**Succinyl-CoA ligase [ADP/GDP-forming] subunit alpha, mitochondrial**	P53597	SUCLG1	3.780E-01	-1.889E-01	3.729E-03	-6.037E-01
**Synapsin-1**	P17600	SYN1	9.636E-02	-5.644E-01	4.555E-06	-1.748E+00
**Synapsin-2**	A0A087WW96	SYN2	8.219E-01	9.930E-02	3.351E-03	-1.330E+00
**Synaptic vesicle membrane protein VAT-1 homolog-like**	Q9HCJ6	VAT1L	6.359E-01	-7.890E-02	2.520E-04	-8.243E-01
**Syntaxin-12 (Fragment)**	B1AJQ6	STX12	9.274E-03	-2.769E-01	1.068E-04	-7.555E-01
**Tensin-1**	Q9HBL0	TNS1	1.779E-01	-2.266E-01	7.246E-03	-8.281E-01
**Tetratricopeptide repeat protein 1**	Q99614	TTC1	1.771E-01	2.199E-01	1.972E-03	-6.709E-01
**Thy-1 membrane glycoprotein (Fragment)**	E9PNQ8	THY1	3.737E-01	-1.813E-01	3.449E-03	-6.902E-01
**Toll-interacting protein**	Q9H0E2	TOLLIP	5.249E-02	-3.263E-01	5.378E-07	-1.067E+00

**Transcriptional activator protein Pur-alpha**	Q00577	PURA	6.072E-01	-5.882E-02	6.402E-05	-6.753E-01
**Triosephosphate isomerase**	P60174	TPI1	7.752E-01	-3.350E-02	3.761E-03	-4.265E-01
**Tubulin alpha-4A chain**	P68366	TUBA4A	1.074E-01	-4.295E-01	9.106E-05	-1.200E+00
**Ubiquitin carboxyl-terminal hydrolase isozyme L1**	P09936	UCHL1	1.749E-01	-1.295E-01	3.159E-06	-7.171E-01
**V-type proton ATPase subunit B, brain isoform**	P21281	ATP6V1B2	2.509E-01	-1.341E-01	2.944E-03	-4.570E-01
**V-type proton ATPase subunit G 2**	O95670	ATP6V1G2	4.878E-02	-3.656E-01	1.212E-03	-7.603E-01
**Vesicle-associated membrane protein-associated protein B/C**	O95292	VAPB	1.291E-01	-2.739E-01	7.900E-03	-5.971E-01
**Visinin-like protein 1**	P62760	VSNL1	2.632E-01	1.893E-01	8.827E-03	-5.201E-01
**Voltage-dependent anion-selective channel protein 1**	P21796	VDAC1	8.931E-02	-1.959E-01	8.184E-04	-4.582E-01
**Voltage-dependent anion-selective channel protein 2**	P45880	VDAC2	5.764E-01	8.214E-02	3.681E-03	-5.328E-01
**10 kDa heat shock protein, mitochondrial**	P61604	HSPE1	2.193E-03	-4.621E-01	1.186E-02	-6.116E-01
**2-oxoglutarate dehydrogenase, mitochondrial**	E9PDF2	OGDH	2.176E-04	-8.500E-01	2.008E-01	-2.373E-01
**40S ribosomal protein S25**	P62851	RPS25	6.343E-03	-7.300E-01	4.216E-01	-1.724E-01
**ADP/ATP translocase 3**	P12236	SLC25A6	8.759E-04	-1.390E+00	3.015E-02	-8.372E-01
**Charged multivesicular body protein 5**	Q9NZZ3	CHMP5	5.335E-03	-6.306E-01	1.737E-01	-3.960E-01
**Disco-interacting protein 2 homolog B**	Q9P265	DIP2B	4.880E-03	-4.825E-01	1.350E-02	-4.604E-01
**Fructose-bisphosphate aldolase**	J3KPS3	ALDOA	3.840E-03	-3.891E-01	9.201E-01	-2.069E-02
**Isoform 4 of Serine protease HTRA2, mitochondrial**	O43464-4	HTRA2	2.254E-03	-7.012E-01	1.171E-01	-3.394E-01
**Liprin-alpha-1**	Q13136	PPFIA1	3.818E-03	-1.001E+00	1.730E-01	-4.482E-01
**Peroxiredoxin-5, mitochondrial**	P30044	PRDX5	7.279E-04	-3.912E-01	5.108E-02	-3.232E-01
**Phosphatidylinositol transfer protein beta isoform**	A0A0A0MSW4	PITPNB	6.055E-04	-1.162E+00	5.537E-01	-1.932E-01
**Tight junction protein 1 (Zona occludens 1), isoform CRA_a**	G3V1L9	TJP1	3.230E-04	-3.909E-01	3.990E-01	9.688E-02
**1-phosphatidylinositol 4,5-bisphosphate phosphodiesterase delta-3**	Q8N3E9	PLCD3	8.400E-03	4.528E-01	1.206E-01	5.074E-01
**Amino acid transporter**	A0A087WT87	SLC1A3	3.972E-03	1.656E+00	6.655E-02	1.194E+00
**Annexin A7**	P20073	ANXA7	5.272E-03	1.620E+00	4.532E-01	3.957E-01
**Nucleobindin-1**	Q02818	NUCB1	2.175E-03	1.041E+00	1.304E-02	1.227E+00

**Peptidyl-prolyl cis-trans isomerase-like 1**	Q9Y3C6	PPIL1	1.882E-03	4.966E-01	4.869E-01	-1.807E-01
**Proteasome subunit alpha type-7**	O14818	PSMA7	4.769E-03	6.311E-01	7.594E-02	3.148E-01
**S-formylglutathione hydrolase**	P10768	ESD	6.331E-03	5.408E-01	2.182E-02	6.010E-01
**Switch-associated protein 70**	E7EMB1	SWAP70	3.323E-03	4.002E-01	8.472E-01	4.483E-02
**2,4-dienoyl-CoA reductase, mitochondrial (Fragment)**	E5RFV2	DECR1	8.119E-01	-8.238E-02	2.733E-04	1.784E+00
**26S proteasome non-ATPase regulatory subunit 2**	Q13200	PSMD2	8.714E-02	-3.109E-01	4.078E-03	1.017E+00
**4-trimethylaminobutyraldehyde dehydrogenase**	P49189	ALDH9A1	9.777E-02	2.549E-01	1.163E-04	8.329E-01
**40S ribosomal protein S14**	P62263	RPS14	3.967E-02	3.874E-01	1.547E-04	9.716E-01
**6-phosphogluconate dehydrogenase, decarboxylating**	P52209	PGD	7.831E-01	-5.604E-02	3.357E-04	1.281E+00
**ATP-dependent RNA helicase DDX19A**	F6QDS0	DDX19A	1.198E-01	-4.580E-01	5.408E-03	1.023E+00
**Acid ceramidase**	E7EMM4	ASAH1	1.167E-02	7.419E-01	2.077E-05	1.630E+00
**Actin-related protein 2/3 complex subunit 5**	O15511	ARPC5	1.620E-02	4.865E-01	4.024E-05	6.331E-01
**Acylamino-acid-releasing enzyme (Fragment)**	H7C393	APEH	4.385E-01	-1.474E-01	2.045E-03	5.897E-01
**Adenylyl cyclase-associated protein 1**	Q01518	CAP1	2.630E-02	4.372E-01	6.176E-04	7.583E-01
**Adseverin**	Q9Y6U3	SCIN	1.071E-02	5.156E-01	5.705E-04	8.284E-01
**Aflatoxin B1 aldehyde reductase member 2 (Fragment)**	H3BLU7	AKR7A2	1.460E-02	3.819E-01	7.476E-03	8.288E-01
**Alpha-1-antitrypsin**	A0A024R6I7	SERPINA1	1.527E-01	9.143E-01	1.739E-03	1.759E+00
**Alpha-1B-glycoprotein**	P04217	A1BG	8.026E-01	8.562E-02	3.396E-03	1.167E+00
**Alpha-aminoadipic semialdehyde dehydrogenase**	P49419	ALDH7A1	5.467E-01	8.011E-02	1.516E-03	4.537E-01
**Alpha-crystallin B chain**	P02511	CRYAB	3.870E-01	2.053E-01	3.905E-03	8.500E-01
**Amine oxidase [flavin-containing] B**	P27338	MAOB	1.609E-02	4.309E-01	1.558E-04	6.326E-01
**Annexin A1**	P04083	ANXA1	4.497E-01	2.204E-01	2.711E-04	1.394E+00
**Apolipoprotein A-I**	P02647	APOA1	4.637E-01	2.115E-01	2.970E-05	1.511E+00
**Apolipoprotein A-II**	V9GYM3	APOA2	5.951E-01	1.570E-01	3.080E-03	1.216E+00
**Apolipoprotein D**	P05090	APOD	1.437E-01	3.843E-01	9.257E-04	8.889E-01
**Apolipoprotein E**	P02649	APOE	3.637E-01	1.311E-01	1.843E-04	7.054E-01
**Apoptosis-inducing factor 1, mitochondrial**	O95831	AIFM1	4.023E-01	6.948E-02	9.616E-04	3.875E-01

**B-cell receptor-associated protein 31**	P51572	BCAP31	1.545E-02	3.932E-01	5.116E-03	4.412E-01
**Band 4.1-like protein 2**	O43491	EPB41L2	8.158E-01	-7.887E-02	1.553E-03	1.424E+00
**Cadherin-2**	P19022	CDH2	3.278E-02	8.871E-01	1.046E-04	1.662E+00
**Calcyphosin**	K7EL21	CAPS	3.187E-01	3.625E-01	8.584E-04	1.525E+00
**Calpain small subunit 1**	A0A0C4DGQ5	CAPNS1	1.642E-01	3.051E-01	4.284E-03	9.525E-01
**Calponin-3**	Q15417	CNN3	5.542E-01	3.522E-01	2.813E-03	1.578E+00
**Catalase**	P04040	CAT	1.427E-01	3.443E-01	2.329E-03	8.906E-01
**Centrosomal protein of 170 kDa**	Q5SW79	CEP170	3.157E-01	1.124E-01	1.351E-04	5.897E-01
**Ceruloplasmin**	P00450	CP	5.680E-01	2.312E-01	4.775E-03	1.257E+00
**Charged multivesicular body protein 4b**	Q9H444	CHMP4B	9.944E-02	2.440E-01	1.780E-04	6.813E-01
**Chloride intracellular channel protein 1**	O00299	CLIC1	2.876E-01	2.553E-01	1.287E-04	1.276E+00
**Copper chaperone for superoxide dismutase**	J3KNF4	CCS	1.434E-01	3.309E-01	1.099E-03	8.716E-01
**Coronin-1C**	Q9ULV4	CORO1C	8.355E-01	-5.384E-02	1.660E-03	1.153E+00
**Cystatin-B**	P04080	CSTB	5.769E-01	7.009E-02	7.087E-03	4.019E-01
**Cytosol aminopeptidase**	P28838	LAP3	2.047E-01	1.843E-01	2.332E-03	4.730E-01
**Cytosolic 10-formyltetrahydrofolate dehydrogenase**	O75891	ALDH1L1	3.026E-01	2.324E-01	4.156E-03	7.964E-01
**D-3-phosphoglycerate dehydrogenase**	O43175	PHGDH	7.928E-02	1.663E-01	1.391E-03	3.997E-01
**Delta-aminolevulinic acid dehydratase**	P13716	ALAD	6.364E-02	3.716E-01	3.834E-03	6.570E-01
**Disintegrin and metalloproteinase domain-containing protein 10**	O14672	ADAM10	1.419E-01	5.797E-01	6.233E-04	1.508E+00
**DnaJ homolog subfamily A member 2**	O60884	DNAJA2	8.592E-01	2.486E-02	2.608E-06	8.054E-01
**DnaJ homolog subfamily B member 1**	P25685	DNAJB1	7.885E-01	-5.496E-02	3.351E-07	1.859E+00
**Erythrocyte band 7 integral membrane protein**	P27105	STOM	4.143E-02	5.612E-01	4.069E-04	1.212E+00
**Estradiol 17-beta-dehydrogenase 8**	Q92506	HSD17B8	4.165E-01	-1.269E-01	1.534E-03	7.109E-01
**Ethylmalonyl-CoA decarboxylase**	Q9NTX5	ECHDC1	2.755E-02	3.735E-01	7.784E-03	5.160E-01
**Extracellular superoxide dismutase [Cu-Zn]**	P08294	SOD3	4.850E-01	2.718E-01	5.033E-03	1.408E+00
**F-box only protein 2**	Q9UK22	FBXO2	3.557E-01	2.114E-01	7.253E-03	1.240E+00
**Flavin reductase (NADPH)**	P30043	BLVRB	7.125E-02	1.336E-01	6.874E-03	3.992E-01
**Fumarylacetoacetase**	P16930	FAH	9.911E-01	-3.176E-03	4.173E-03	8.653E-01

**Galectin-3**	P17931	LGALS3	7.406E-01	5.522E-02	5.953E-03	5.208E-01
**Glia maturation factor beta (Fragment)**	G3V4P8	GMFB	7.137E-01	-4.899E-02	3.788E-03	4.422E-01
**Glutathione S-transferase Mu 2**	P28161	GSTM2	4.574E-01	1.232E-01	3.428E-03	8.850E-01
**Glutathione S-transferase P**	P09211	GSTP1	4.543E-01	8.822E-02	9.080E-03	4.147E-01
**Hemoglobin subunit alpha**	P69905	HBA1	6.410E-02	5.821E-01	1.716E-04	1.414E+00
**Hemoglobin subunit beta**	P68871	HBB	4.832E-01	2.394E-01	8.632E-03	1.125E+00
**Hemoglobin subunit delta**	P02042	HBD	3.504E-01	2.626E-01	5.235E-04	1.432E+00
**Hemopexin**	P02790	HPX	4.123E-01	3.227E-01	3.701E-03	1.383E+00
**Histamine N-methyltransferase**	P50135	HNMT	8.305E-01	6.994E-02	5.375E-03	7.872E-01
**Hsc70-interacting protein**	P50502	ST13	1.674E-01	1.855E-01	1.635E-03	5.079E-01
**Hydroxyacyl-coenzyme A dehydrogenase, mitochondrial**	A0A0A0MSE2	HADH	9.286E-01	3.001E-02	4.720E-03	1.147E+00
**Integrin alpha-6**	P23229	ITGA6	2.150E-01	1.754E-01	5.429E-05	9.955E-01
**Intercellular adhesion molecule 1**	P05362	ICAM1	8.858E-01	3.290E-02	3.708E-03	7.018E-01
**Isoform 2 of 40S ribosomal protein S3**	P23396-2	RPS3	1.420E-02	5.042E-01	5.916E-03	8.091E-01
**Isoform 2 of Annexin A2**	P07355-2	ANXA2	3.407E-01	1.759E-01	7.923E-06	1.233E+00
**Isoform 2 of Filamin-C**	Q14315-2	FLNC	2.036E-01	2.357E-01	1.444E-04	1.077E+00
**Isoform 2 of N-acetyl-D-glucosamine kinase**	Q9UJ70-2	NAGK	9.127E-01	2.350E-02	2.102E-03	7.846E-01
**Isoform 2 of Sorcin**	P30626-2	SRI	3.527E-02	4.760E-01	5.071E-03	8.651E-01
**Isoform 2 of Transgelin-2**	P37802-2	TAGLN2	3.297E-01	2.751E-01	2.507E-03	9.969E-01
**Isoform 3 of Ankyrin-3**	Q12955-5	ANK3	5.593E-01	8.421E-02	7.390E-04	6.928E-01
**Isoform 3 of Gamma-glutamyltransferase 5**	P36269-3	GGT5	1.240E-01	-4.468E-01	2.462E-03	9.958E-01
**Isoform 4 of Integrin beta-1**	P05556-4	ITGB1	4.109E-01	1.129E-01	4.786E-06	9.852E-01
**Isoform 4 of Non-specific lipid-transfer protein**	P22307-4	SCP2	4.420E-01	9.641E-02	4.072E-03	5.398E-01
**Isoform 4 of Plasminogen activator inhibitor 1 RNA-binding protein**	Q8NC51-4	SERBP1	2.784E-02	6.295E-01	2.448E-03	1.015E+00
**Isoform 5 of CD44 antigen**	P16070-5	CD44	2.838E-01	2.290E-01	9.365E-04	7.852E-01
**Isoform L-MPZ of Myelin protein P0**	P25189-2	MPZ	2.913E-02	1.020E+00	8.289E-05	2.834E+00
**Isoform LCRMP-4 of Dihydropyrimidinase-related protein 3**	Q14195-2	DPYSL3	1.203E-01	1.876E-01	9.339E-04	5.250E-01

**L-aminoadipate-semialdehyde dehydrogenase-phosphopantetheinyl transferase**	Q9NRN7	AASDHPPT	7.044E-01	1.063E-01	1.056E-04	1.663E+00
**Lethal(2) giant larvae protein homolog 1**	Q15334	LLGL1	9.858E-01	4.357E-03	8.963E-03	7.322E-01
**Leukotriene A-4 hydrolase**	P09960	LTA4H	2.645E-01	1.913E-01	5.959E-04	8.415E-01
**Malignant T-cell-amplified sequence 1**	Q9ULC4	MCTS1	4.645E-01	1.963E-01	8.348E-03	6.082E-01
**Microtubule-associated protein RP/EB family member 1**	Q15691	MAPRE1	4.444E-01	-1.229E-01	2.201E-03	9.661E-01
**Mitochondrial carrier homolog 2**	Q9Y6C9	MTCH2	2.263E-01	2.385E-01	2.069E-03	7.053E-01
**Myelin proteolipid protein**	P60201	PLP1	3.122E-02	1.126E+00	8.629E-03	1.304E+00
**Nestin**	P48681	NES	5.171E-01	-1.055E-01	2.787E-03	8.322E-01
**Neural cell adhesion molecule 1**	P13591	NCAM1	4.571E-02	6.019E-01	3.004E-03	1.064E+00
**Neuroblast differentiation-associated protein AHNAK**	Q09666	AHNAK	1.187E-01	3.325E-01	5.694E-04	8.831E-01
**Neutral alpha-glucosidase AB**	Q14697	GANAB	1.032E-02	4.926E-01	4.523E-03	6.368E-01
**Neutrophil defensin 3**	P59666	DEFA3	2.894E-01	2.471E-01	3.034E-04	8.416E-01
**Nidogen-2**	Q14112	NID2	4.045E-01	-9.650E-02	4.093E-03	4.423E-01
**Nucleolin**	P19338	NCL	8.495E-01	-2.417E-02	2.669E-05	6.411E-01
**PDZ and LIM domain protein 5**	Q96HC4	PDLIM5	9.006E-02	4.313E-01	1.485E-03	7.535E-01
**PHD finger-like domain-containing protein 5A**	Q7RTV0	PHF5A	5.254E-02	2.964E-01	3.699E-03	9.681E-01
**Palladin**	Q8WX93	PALLD	1.697E-01	3.668E-01	6.061E-03	7.896E-01
**Periaxin**	Q9BXM0	PRX	2.493E-01	6.065E-01	1.548E-03	2.719E+00
**Phosphoserine aminotransferase**	Q9Y617	PSAT1	1.280E-01	2.366E-01	1.362E-03	5.728E-01
**Polypyrimidine tract binding protein 1, isoform CRA_b**	A6NLN1	PTBP1	6.204E-01	1.824E-01	4.115E-03	1.052E+00
**Profilin**	G5E9Q6	PFN2	4.298E-01	1.646E-01	3.615E-04	8.857E-01
**Profilin-2**	P35080	PFN2	2.738E-01	2.039E-01	2.199E-03	9.422E-01
**Prolargin**	P51888	PRELP	1.377E-01	6.757E-01	2.007E-03	1.855E+00
**Protein BRICK1**	Q8WUW1	BRK1	4.975E-01	-1.106E-01	6.743E-03	6.311E-01
**Protein CutA**	O60888	CUTA	1.622E-01	-2.337E-01	1.495E-03	6.517E-01
**Protein S100-A11**	P31949	S100A11	2.674E-01	-6.314E-01	5.162E-05	2.076E+00
**Protein S100-A6**	P06703	S100A6	2.945E-01	3.704E-01	1.083E-03	1.421E+00
**Protein disulfide-isomerase**	P07237	P4HB	5.051E-02	4.850E-01	1.052E-03	1.069E+00
**Protein enabled homolog**	Q8N8S7	ENAH	9.880E-01	1.988E-03	3.267E-04	5.757E-01
**S-adenosylmethionine synthase isoform type-2**	P31153	MAT2A	9.151E-01	-1.110E-02	6.753E-03	4.174E-01

**Selenium-binding protein 1**	Q13228	SELENBP1	7.574E-01	9.302E-02	1.173E-03	8.721E-01
**Septin 11, isoform CRA_b**	D6RGI3	SEPT11	6.912E-01	-6.688E-02	6.020E-04	7.254E-01
**Serum albumin**	P02768	ALB	9.739E-02	3.416E-01	8.722E-06	1.035E+00
**Spectrin beta chain, non-erythrocytic 2**	O15020	SPTBN2	7.538E-01	4.843E-02	4.674E-03	4.962E-01
**Superoxide dismutase [Mn], mitochondrial**	P04179	SOD2	2.639E-01	3.495E-01	2.307E-03	9.317E-01
**Talin-1**	Q9Y490	TLN1	8.608E-02	2.392E-01	7.330E-05	5.514E-01
**Transcription factor BTF3**	P20290	BTF3	2.447E-02	3.289E-01	4.595E-03	5.035E-01
**Transgelin**	Q01995	TAGLN	4.405E-02	8.485E-01	6.366E-03	1.107E+00
**Transitional endoplasmic reticulum ATPase**	P55072	VCP	2.100E-01	1.783E-01	1.435E-04	7.130E-01
**Transthyretin**	P02766	TTR	1.251E-01	4.396E-01	4.826E-05	1.373E+00
**Tropomyosin alpha-4 chain**	P67936	TPM4	2.513E-01	2.479E-01	4.375E-04	7.180E-01
**Vinculin**	P18206	VCL	1.772E-01	2.511E-01	1.228E-03	7.727E-01
**6-phosphogluconolactonase**	O95336	PGLS	2.665E-03	4.019E-01	2.739E-04	6.230E-01
**Moesin**	P26038	MSN	8.651E-03	4.031E-01	3.413E-03	4.704E-01
**Small glutamine-rich tetratricopeptide repeat-containing protein alpha**	O43765	SGTA	4.064E-03	4.313E-01	1.679E-03	5.905E-01
**Alcohol dehydrogenase class-3**	P11766	ADH5	7.339E-03	5.509E-01	2.710E-03	5.822E-01
**Annexin A5**	P08758	ANXA5	7.051E-03	6.099E-01	2.823E-03	1.117E+00
**ATP-dependent 6-phosphofructokinase, muscle type**	P08237	PFKM	3.140E-04	6.280E-01	1.519E-05	6.044E-01
**Heat shock protein beta-8**	Q9UJY1	HSPB8	8.195E-03	7.376E-01	3.868E-03	8.926E-01
**Carbonic anhydrase 1**	P00915	CA1	9.385E-03	8.815E-01	1.850E-03	1.610E+00
**Guanine nucleotide-binding protein G(i) subunit alpha-2**	P04899	GNAI2	1.104E-03	1.052E+00	3.952E-03	1.146E+00

Supplementary Table S2: Shows the 21 Ingenuity pathways that were found to be significantly regulated (-log p-value >1.3) when comparing ALS and FTLD-U with control subjects. The column named Molecules contains the gene names for the molecules found significantly regulated involved in each enriched pathway.

Ingenuity Canonical Pathways	ALS		FTLD-U	
	-log(p-value)	Molecules	-log(p-value)	Molecules
Mitochondrial Dysfunction	27.50	HSD17B10,NDUFA4,SDHB,ATP5H,NDUFA7,UQCRH,COX5B,ACO2,NDUFA2,ATP5E,VDAC2,MAOB,NDUFA5,NDUFS1,SOD2,NDUFS6,ATP5I,ATP5F1,NDUFA8,NDUFS4,NDUFV1,COX6B1,ATP5A1,ATP5O,NDUFS5,NDUFV2,NDUFS8,CAT,UQCRC2,COX5A,CYCS,VDAC1,UQCRC1	21.3	NDUFA4,ATP5H,NDUFA7,UQCRH,PRDX5,COX5B,COX7C,NDUFA2,NDUFS5,NDUFA5,MAOB,NDUFS1,NDUFS6,HTRA2,GPX4,OGDH,ATP5I,ATP5F1,NDUFA8

Oxidative Phosphorylation	24.20	NDUFA4,NDUFV1,COX6B1,SDHB,ATP5H,NDUFA7,UQCRH,COX5B,ATP5A1,ATP5O,NDUFA2,ATP5E,NDUFS5,NDUFS1,NDUFA5,NDUFV2,NDUFS8,UQCRC2,NDUFS6,COX5A,CYCS,UQCRC1,ATP5I,ATP5F1,NDUFS4,NDUFA8	16.6	NDUFS5,NDUFA4,NDUFS1,NDUFA5,ATP5H,NDUFA7,UQCRH,COX5B,COX7C,NDUFS6,ATP5I,ATP5F1,NDUFA2,NDUFA8
TCA Cycle II (Eukaryotic)	7.72	SDHB,SUCLG1,ACO2,DLD,MDH2,FH,MDH1	2.38	OGDH,MDH2
Gluconeogenesis I	7.44	GPI,ENO2,ALDOA,PGAM2,MDH2,MDH1,ALDOC	2.3	ALDOA,MDH2
Glycolysis I	7.44	GPI,TPI1,ENO2,ALDOA,PGAM2,ALDOC,PFKM	2.3	ALDOA,PFKM
Aspartate Degradation II	5.91	GOT1,GOT2,MDH2,MDH1	1.53	MDH2,
NRF2-mediated Oxidative Stress Response	5.06	SOD2,GSTM2,MAPK1,STIP1,VCP,CAT,HSPB8,DNAJA2,TXN,GSTO1,SOD3,GSTP1	3	FTL,HSPB8,SOD1,MGST3,FTH1
Glutaryl-CoA Degradation	4.78	HSD17B10,CA1,HADH,HSD17B8	1.31	CA1,
Xenobiotic Metabolism Signaling	3.90	ALDH1L1,MAOB,GSTM2,MAPK1,PPP2R4,CAT,GSTO1,SOD3,GSTP1,ALDH9A1,ESD,CAMK2B,ALDH7A1	1.54	FTL,MAOB,MGST3,ESD
Superoxide Radicals Degradation	3.85	SOD2,CAT,SOD3	1.48	SOD1,
Noradrenaline and Adrenaline Degradation	3.76	HSD17B10,ADH5,MAOB,ALDH9A1,ALDH7A1	1.95	ADH5,MAOB
Formaldehyde Oxidation II (Glutathione-dependent)	3.71	ADH5,ESD	4.75	ADH5,ESD
Sucrose Degradation V (Mammalian)	3.68	TPI1,ALDOA,ALDOC	1.43	ALDOA,
Pentose Phosphate Pathway (Oxidative Branch)	2.94	PGD,PGLS	1.78	PGLS,
Serotonin Degradation	2.62	HSD17B10,ADH5,MAOB,ALDH9A1,ALDH7A1	1.49	ADH5,MAOB
Aldosterone Signaling in Epithelial Cells	2.26	PLCD1,CRYAB,MAPK1,HSPE1,HSPB8,HSPA4L,HSPA12A	3.33	PLCD1,PLCD3,HSPE1,HSPB8,HSPA2
Pentose Phosphate Pathway	2.94	PGD,PGLS	1.78	PGLS,
p70S6K Signaling	1.59	PLCD1,YWHAH,MAPK1,PPP2R4,BCAP31	1.85	PLCD1,PLCD3,BCAP31
Protein Ubiquitination Pathway	1.57	UCHL1,CRYAB,HSPE1,HSPB8,PSMA1,SKP1,HSPA4L,HSPA12A	2.34	PSMA7,HSPE1,HSPB8,SKP1,HSPA2
UVA-Induced MAPK Signaling	1.46	PLCD1,MAPK1,CYCS,PARP1	2.21	PLCD1,PLCD3,PARP1
Antiproliferative Role of TOB in T Cell Signaling	1.30	MAPK1,SKP1	2.27	PABPC1,SKP1

Supplementary Table S3: Shows the ideal conditions for the western blot employed to analyze each target under study. Antibody dilutions (v-v) for the primary and secondary antibodies, acrylamide percentages for the pre-casted gels and blocking and antibody dilution solution.

Western blot	Primary antibody dilution	Secondary antibody dilution	Acrilamide %	Blocking and Antibody dilution solution.
LGALS3	1-1000	anti-mouse 1-5000	4-15%	5 % milk
TTR	1-1000	anti-sheep 1-3000	4-15%	5 % milk
SELENBP1	1-1000	anti-rabbit 1-5000	10-20%	5 % milk
Pin-1	1-1000	anti-rabbit 1-5000	10-20%	5 % BSA
S100A11	1-100	anti-rabbit 1-5000	10-20%	5 % milk
S100A6	1-1000	anti-rabbit 1-5000	10-20%	5 % BSA
CACYBP	1-1000	anti-rabbit 1-5000	10-20%	5 % BSA
ROCK 2	1-10000	anti-rabbit 1-5000	10-20%	5 % milk
PHB1	1-1000	anti-rabbit 1-5000	10-20%	5 % BSA
PHB2	1-1000	anti-rabbit 1-5000	10-20%	5 % BSA
AKT	1-1000	anti-rabbit 1-5000	10-20%	5 % BSA
pAKT	Jan-00	anti-rabbit 1-5000	10-20%	5 % BSA
FAK	1-1000	anti-rabbit 1-5000	10-20%	5 % BSA
pFAK	1-1000	anti-rabbit 1-5000	10-20%	5 % BSA
ERK	1-1000	anti-rabbit 1-5000	10-20%	5 % BSA
pERK	Jan-00	anti-rabbit 1-5000	10-20%	5 % BSA
MEK	1-1000	anti-rabbit 1-5000	10-20%	5 % BSA
pMEK	1-1000	anti-rabbit 1-5000	10-20%	5 % BSA
